# Gene expression pattern of functional neuronal cells derived from human bone marrow mesenchymal stromal cells

**DOI:** 10.1186/1471-2164-9-166

**Published:** 2008-04-11

**Authors:** Tatiana Tondreau, Marielle Dejeneffe, Nathalie Meuleman, Basile Stamatopoulos, Alain Delforge, Philippe Martiat, Dominique Bron, Laurence Lagneaux

**Affiliations:** 1 Institut Jules Bordet, Université Libre de Bruxelles (ULB), Laboratory of Experimental Hematology, 121, Bd de Waterloo, 1000 Brussels, Belgium

## Abstract

**Background:**

Neuronal tissue has limited potential to self-renew or repair after neurological diseases. Cellular therapies using stem cells are promising approaches for the treatment of neurological diseases. However, the clinical use of embryonic stem cells or foetal tissues is limited by ethical considerations and other scientific problems. Thus, bone marrow mesenchymal stomal cells (BM-MSC) could represent an alternative source of stem cells for cell replacement therapies. Indeed, many studies have demonstrated that MSC can give rise to neuronal cells as well as many tissue-specific cell phenotypes.

**Methods:**

BM-MSC were differentiated in neuron-like cells under specific induction (NPBM + cAMP + IBMX + NGF + Insulin). By day ten, differentiated cells presented an expression profile of real neurons. Functionality of these differentiated cells was evaluated by calcium influx through glutamate receptor AMPA3.

**Results:**

Using microarray analysis, we compared gene expression profile of these different samples, before and after neurogenic differentiation. Among the 1943 genes differentially expressed, genes down-regulated are involved in osteogenesis, chondrogenesis, adipogenesis, myogenesis and extracellular matrix component (tuftelin, AGC1, FADS3, tropomyosin, fibronectin, ECM2, HAPLN1, vimentin). Interestingly, genes implicated in neurogenesis are increased. Most of them are involved in the synaptic transmission and long term potentialisation as cortactin, CASK, SYNCRIP, SYNTL4 and STX1. Other genes are involved in neurite outgrowth, early neuronal cell development, neuropeptide signaling/synthesis and neuronal receptor (FK506, ARHGAP6, CDKRAP2, PMCH, GFPT2, GRIA3, MCT6, BDNF, PENK, amphiregulin, neurofilament 3, Epha4, synaptotagmin). Using real time RT-PCR, we confirmed the expression of selected neuronal genes: NEGR1, GRIA3 (AMPA3), NEF3, PENK and Epha4. Functionality of these neuron-like cells was demonstrated by Ca^2+ ^influx through glutamate receptor channel (AMPA3) in the presence of two agonist glutamate, AMPA or CNQX antagonist.

**Conclusion:**

Our results demonstrate that BM-MSC have the potential to differentiate in neuronal cells with specific gene expression and functional properties. BM-MSC are thus promising candidates for cell-based therapy of neurodegenerative diseases

## Background

Neurodegenerative disorders, such as Parkinson's and Alzheimer's disease, stroke, epilepsy or trauma are characterized by a loss of neurons. Neural tissue has a limited capacity of repair after injury and neural stem cells are localized to a selected region. Different studies have proposed the use of fetal neural stem cells or neural cells derived from embryonic stem cells to treat brain injury [1,2]. However, their use is limited by ethical considerations and other scientific problems.

It has been demonstrated that mesenchymal stromal cells (MSC) may play a role in neurogenesis or repair brain injury [3-5]. Expanded MSC were injected in different parts of the brain (striatum or spinal cord) in a mouse model of Parkinson's disease, albino-rat or injured spinal cord rat. From five days to 5 weeks post-injection the migration of MSC and the expression of typical markers for neurons or astrocytes were observed. MSC can also promote the neuronal recovery [6]. Using co-cultures with mesencephalic cells, hippocampal brain slice or cerebellar granule neurons, some investigators have demonstrated the differentiation of bone marrow-MSC (BM-MSC) into cells expressing neuronal/glial proteins. These results suggest that cell-cell contacts play an important role in neural differentiation [7,8]. However, in these both cases (graft and coculture) the fusion between MSC and neuron/glial cells cannot be excluded.

In vitro the neural differentiation of MSC documented in literature required 5 hours to 10 days to obtain cells with morphologic and expression patterns similar to "real" neurons [9,10]. However typical morphology observed with long thin neuritis like neurons seems associated with chemical cytotoxicity induced by the neurogenic medium. In fact, the use of Dimethyl Sulfoxide (DMSO), Beta-Mercaptoethanol (BME) or Butyl Hydroxyanisole (BHA) may cause retraction of the cytoplasm due to a rapid disruption of actin cytoskeleton [11,12]. On the other hand, the expression of neuronal markers evaluated by immunolabeling was not always increased and was not every time confirmed by RT-PCR analysis or proteomic analysis [13].

As confirm by other group, we have previously demonstrated that before any differentiation MSC can express specific neural proteins [14,15]. Induced cells with Neuronal Progenitor Basal Medium (NPBM medium) or Neurobasal added with a mix cytokines, demonstrate an increased expression of Tyrosine Hydroxylase (TH), MAP-2, NF, NeuN, GABA, nestin and GFAP [14,16].

Although specific protein expression has been evaluated, little is known about the biological process, the molecular functions and the gene modulation during MSC differentiation process.

In this study, to confirm the differentiation of MSC to neural phenotype and to demonstrate that this differentiation is not an artifact, we performed microarray analysis to establish the gene expression profile of MSC before and after neurogenic differentiation. Among the 54000 probe sets corresponding to 39500 genes, 1943 probe sets were modulated with a fold change of 1,5. Quantitative real time PCR was also performed to confirm microarray data. In this study, several neurogenic genes were upregulated.

Our results provide the ability of MSC to differentiate into neurons and to evoke a fast synaptic transmission. These results were confirmed by rapid increase level of intracellular Ca ^2+ ^level after glutamate or AMPA stimuli.

## Results and discussion

### Characterization and differentiation capacity of BM-MSC

**Figure 1:**
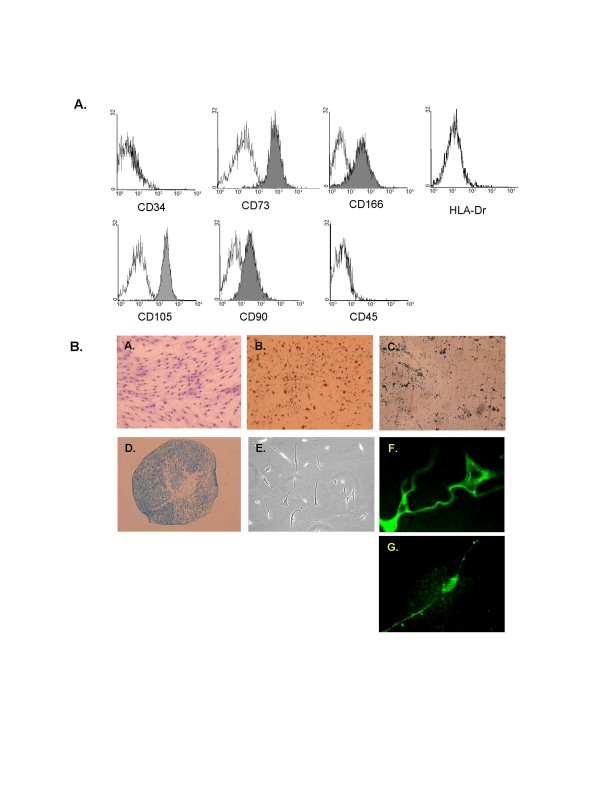
**Characterization and differentiation of BM-MSC**. (A) Reprentative flow cytometric analysis of cultured mesenchymal stomal cells. Solid white represente the isotype control. (B) A. BM-MSC, displayed an homogeneous morphology of fibroblastic cells. Cells were stained with May Grunwald Giemsa staining (40×). Under specific induction BM-MSC were differentiated into (B) adipocytes (lipid vacuoles were colored by Oil Red O, ×40), (C) osteocytes (calcium deposits were revealed by Von Kossa method, ×40), (D) chondrocytes (cell pellet was sectioned and stained by toluidine blue, ×4), and (E) neuron-like cells derived from BM-MSC upon treatment with neurogenic medium (×40). Expression of nestin and MAP-2 after 10 days induction, detected by immunofluorescence (F and G, 100×).

We have previously demonstrated that two passages were required to isolate a homogeneous population of MSC using the plastic adhesion method [17]. These cells displayed spindle-shape morphology and were identified by their expression for CD73, CD105, CD90 but negative for all hematopoietic markers (CD45, CD34, HLA-Dr). Before neurogenic induction, cells were also evaluated for their multilineage capacity. These cells can be induced to differentiate into osteocytes, adipocytes and chondrocytes lineage using specific culture media (Figure 1A,B).

After 2 passages, more than 72% of cells naturally express Nestin. Nevertheless, MAP-2 and TH expression were weaker, less than 5,4% and 12.5% respectively.

**Figure 2:**
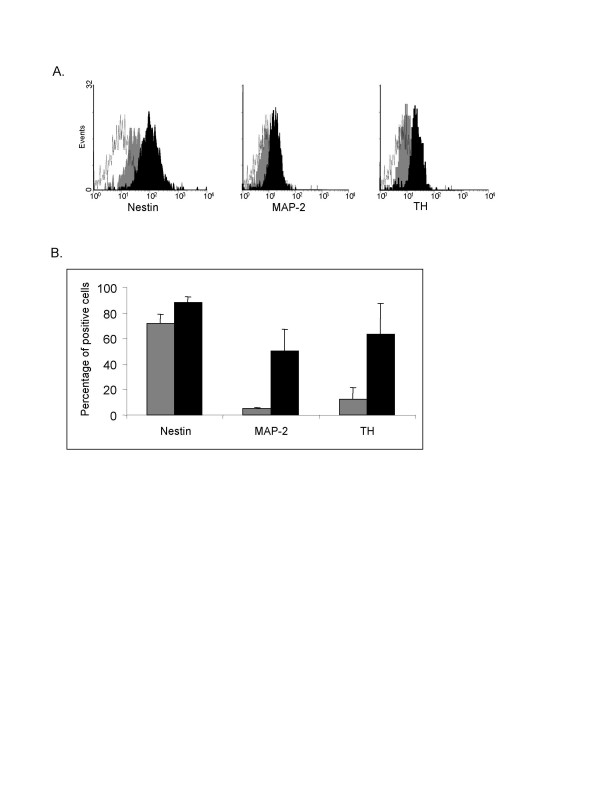
**Flow cytometric analysis of neurone like cells**. Expression of nestin, MAP-2 and TH neural proteins before and after 10 day exposure with neurogenic medium. (A) Typical FACS analysis of neuronal markers expressed by MSC before (gray histogram) and after (black histogram) differentiation. White histogram represents the isotype control. (B) Percentage of positive cells for nestin, MAP-2 and TH before (gray) and after (black) specific neurogenic induction. The mean (SEM) was obtained from three independent experiments.

After 10 days under neurogenic induction, the expression of nestin remains relatively stable, but we observed a dramatically increase of more mature neuronal proteins MAP-2 and TH, with more than 50,1% and 63,1% of positive cells respectively (Figure 2A,B).

### Microarray analysis

In the aim to confirm the neuronal differentiation of BM-MSC and to determine if they can modify their gene expression profile in response to the neurogenic medium, we performed microarray analysis.

For this study, three different samples of BM-MSC were investigated before and after neurogenic differentiation with Affymetrix microarray analysis.

Of 1943 probes sets, a total of 913 upregulated genes and 1030 downregulated genes were significantly modulated after differentiation with a fold change of 1,5.

The identified genes were classified into different groups: neuronal channel/transport, synaptic differentiation/transmission, neuronal development, mesodermal differentiation, extra cellular matrix component, cytoskeleton and cell cycle/proliferation, regarding their function and the pathway which in they were implicated (Table 2) [see additional file 1].

Recent studies demonstrated that treatment of MSC with chemical agents including DMSO/BHA induced a neuronal morphology causing by disruption of cytoskeleton, a loss of cell-adherence but BM-MSC showed signs of death [11,13,19]. In this study, we observed that genes implied in proliferation (SPD35, CDC20, GPC4, CDC42) were downregulated but no variation of gene expression involved in cell apoptosis or death were observed (DAPK3, PDCD2, APAF1, ACIN1). Concordant results were recently described using a similar neurogenic medium. They postulate the post-mitotic status of differentiated cells resulting by down regulation of genes implicated in the cell cycle [20].

Comparing the gene expression profile of human MSC with CD34 positive cells, Silva et al demonstrated that some genes were exclusively or highly represented in MSC (Collagen type I, Fibronectin, MYL9, Biglycan) [18]. We observed that all of these genes are downregulated after neurogenic differentiation. Moreover, the "Mesenchymal stem cell protein DSC96" and the "Mesoderm specific transcript (MEST), two specific genes of mesodermal lineage have an important decrease of 5,8 and 15,2 fold respectively after neurogenic differentiation. A decreased expression of genes involved in osteogenesis, myogenesis or lipid metabolism was also observed. Differentiated cells were not able to mineralize the matrix due to the important decreased of TUFT1, COL _IV, V, XI, XII_, OSF-2 and the modification of the extracellular matrix component after the differentiation (CSPG4, HAPLN1, ECM2, FN1). MSC were first identified for their capacity of differentiation into adipocytes or osteocytes two distinct mesodermal cell lineages. It is well established that the decrease of osteoblast differentiation is associated with increase adipocyte formation. In fact osteoporosis due to the aging correlated with an increased of adipocytes into the bone marrow [19]. We did not observe an increased expression of adipocyte associated genes in response to the downregulation of gene linked to osteogenesis. In contrast, genes involved in adipogenesis are decreased (MGLL, FADS3, LYPLAL1).

**Figure 3:**
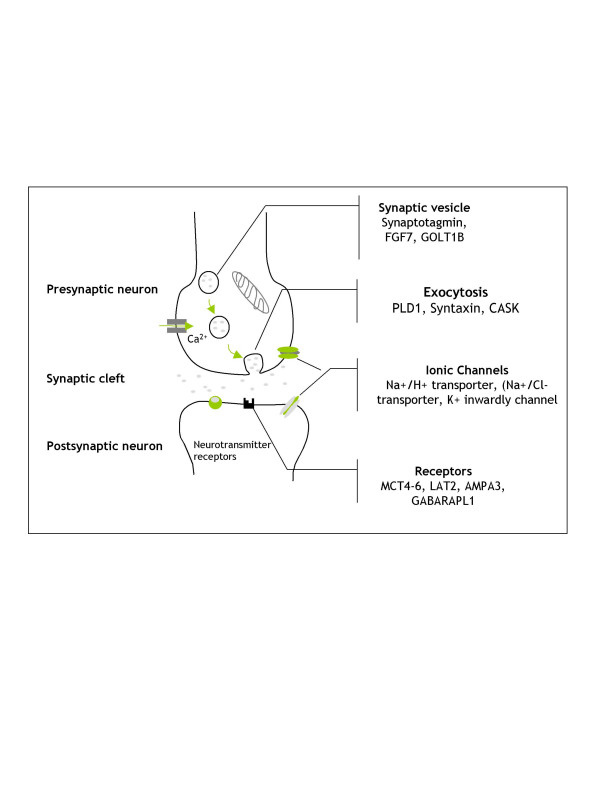
**The synatptic transmission gene expressed**. Schematic representation of localization and role of genes increased after neurogenic induction and involved in the synaptic transmission.

According to the downregulation of mesenchymal genes, differentiated cells have an increased expression of typical neural genes involved in the synaptic transmission, neurite outgrowth, neurotransmitter signaling/receptors and neuronal cell development. Our microarray analysis revealed increased expression of synaptic protein mRNA involved in the synaptic vesicle pathway, such as Synaptotagmin (SYT), Phospholipase D1 (PLD1) Syntaxin 1A (STX1A), Brain derived neurotrophic factor (BDNF) and FGF7, favoring the presynaptic vesicle clustering (Figure 3).

Other genes upregulated after neurogenic induction were involved in (1) **synaptic transmission/long term potentialisation**: Ca2+/Calmodulin (CaM)-dependent kinases (CASK), Promelanin concentrating hormone (PMCH), Choline Transporter (CTL1), Proenkephalin (PENK), (2) **neurite outgrowth and neurogenesis**: Neuronal Growth Regulator 1 (NRG1), Brain abundant membrane attached signal protein 1 (BASP1), Amphiregulin (AREG), Eph receptor A4 (EPHA4), neurofilament 3 (NEF3). (3) **neurotransmitter receptor/transporters and channels**: Glutamate receptor (AMPA3),, Na+/Sialic cotransporter (LAT2), Potassium voltage gated channel, subfamily G, Member 1 (KCNG1), Gaba (A) receptor associated protein-like 1 (GABARAPL1).

### Quantitative RT-PCR analysis

**Figure 4:**
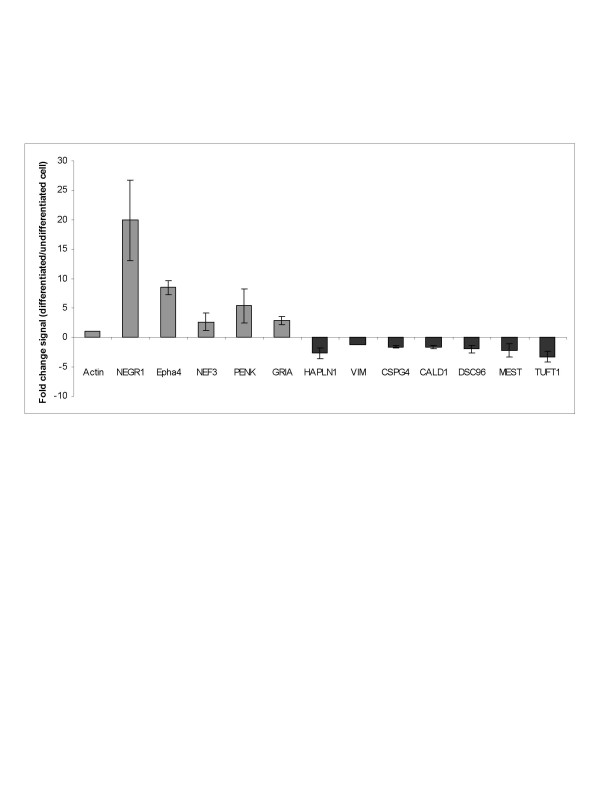
**Quantitative real-time PCR analysis**. Quantitative real-time PCR analysis using β-actin as a housekeeping gene and calibrated with the PANOMICS: Human brain total RNA (invitrogen, Brussels, Belgium). Results are expressed in fold change signal between differentiated and non-differentiated BM-MSC after the neurogenic induction. Selected neuronal genes were NEGR1, EphA4, NEF3, GRIA3 (AMPA), PENK (Grey column). Selected mesenchymal genes were HAPLN1, VIM, CSPG4, CALD1, DSC96, MEST, TUFT1 (black column).

To confirm the gene expression profile revealed by microarray analysis, quantitative RT-PCR was carried out. Representative genes involved into neural development/differentiation (EphA4, AMPA3, NEGR1, NEF3, PENK) and in mesodermal cell lineage (HAPLN1, CSPG4, CALD1, VIM, DSC96, MEST, TUFT1) were selected from our microarray analysis. We evaluated the mRNA gene expression between undifferentiated/differentiated cells in comparison with a housekeeping gene (β-actin). As show in figure 4, using quantitative real-time PCR, we confirmed the increased expression of EphA4, AMPA3, NEGR1, NEF3 and PENK by differentiated MSC ten days after neurogenic induction. In contrast, genes expressed naturally by BM-MSC were downregulated after neurogenic induction (HAPLN1, CSPG4, CALD1, VIM, DSC96, MEST, TUFT1).

### Calcium Influx

Increased mRNA expression of a high number of neural synaptic genes by microarray or real-time PCR by differentiated BM-MSC is not sufficient to demonstrate the differentiation status. It will be important that neural-like cells obtained after in vitro differentiation of BM-MSC were functional and able to have synaptic transmission potential.

Excitory synaptic currents are mediated by different types of glutamate receptor channels including AMPA3, also named GRIA3 or GluR3. Calcium influx plays an important role in the synaptic transmission. In this way, we investigated the Calcium influx though GRIA3 receptor. Ca^2+ ^influx was measured using Fluo-4 NW assay kit in the presence of glutamate (100 μM), or AMPA (40 μM) both agonist of GRIA3 and CNQX (10 μM), antagonist of GRIA3.

**Figure 5:**
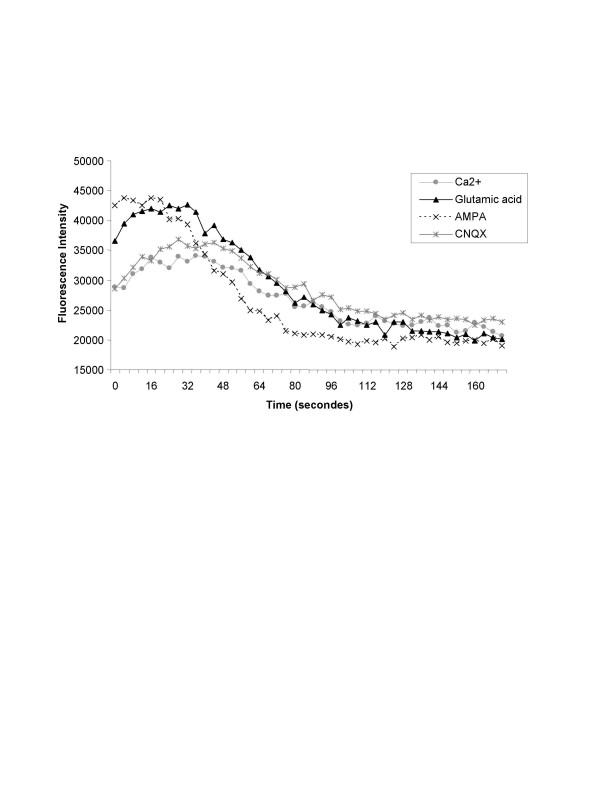
**Intracellular Calcium influx**. Representative variation of intracellular Calcium influx in differentiated MSC treated with AMPA (40 μM, ---×---), glutamate (100 μM, ) or CNQX (10 μM, ). Results are expressed as fluorescence intensity at 4 second-intervals using the Fluo-4 NW tracer. Agonists (AMPA or Glutamate) and antagonist CNQX were compared with spontaneous level of calcium influx in presence of Ca2+ alone ().

Treatment of GRIA3 with AMPA or Glutamate, demonstrated an important increased of Ca^2+ ^influx in comparison with the addition of CNQX, the specific antagonist of GRIA3 (Figure 5) were demonstrated. The presence of CNQX completely abolished the entry of Ca2+ through the AMPA3 receptor.

All measures were tested in a calcium buffer containing 0,15 μM of free Ca^2+^. These results suggest both the fully differentiation of BM-MSC into phenotypical neurons but also their functionality in our neurogenic conditions.

## Discussion

Accordingly to easy accessibility within the bone marrow and the important potential of differentiation, BM-MSC represent interesting candidates for gene therapy, tissue engineering and a wide range of regenerative medicine applications [23]. Recent observations demonstrated the capacity of BM-MSC to migrate and to differentiate into various damaged tissues. However, molecular mechanisms implied in the differentiation process remain largely unknown. A great number of studies have evaluated the neuronal differentiation potential of BM-MSC in vitro, using different protocols of induction. DMSO, RA, BHA, IBMX or BME were used as inducible agents, and morphologic changes of BM-MSC were rapidly observed (within few hours) with variable percentage of cell expressing neuronal markers (neuron-specific enolase, tau, neuronal nuclear antigen, [9,24,25]. Recently, Neuhuber et al, reevaluated the *in vitro *differentiation protocols and demonstrated the chemical toxicity of these agents on the cytoskeleton of MSC. These chemical agents induced rapid disruption of actin with an aberrant expression of neuronal proteins arguing against neurogeneic differentiation [11]. Using microarray analysis genes modulated by DMSO/BHA induction were different from those observed during neurogenesis in neural tissue. Moreover, differentiation process required cascades of transcriptional events that could not be able to realize within a few hours [13,26]. They demonstrated that exposition to DMSO/BHA resulted to a reversible lost cell contact with the substratum leading to the detachment of BM-MSC. In these conditions, the morphological shape seems associated to a decrease of cell anchorage and not to a real neuritis outgrowth. Moreover, a high percentage of apoptotic cells were present after induction with DMSO/BHA. However, dermal fibroblasts also responded to chemical induction by forming long thin process and expressing some neuron and glial markers. The rapidity of neuron-like differentiation argues against physiological cell differentiation [27].

We did not observe morphological changes using our neurogenic induction medium on fibroblastic lineage (MRC5). In comparison with BM-MSC, no expression of neuronal markers was observed during the treatment of MRC5 cells (data not shown). This observation demonstrated the absence of cytotoxic effect on the cytoskeleton in our conditions of neurogenic induction.

The presence or absence of specific antigen expression can not prove the differentiation of MSC. In fact, undifferentiated BM-MSC can express a large number of antigens from other tissues in which they reside [14,28-30].

During the differentiation process and contrasting to previous studies, we did not observed morphological changes within a few hours using our neurogenic induction medium [9,23,31]. An average of four days was required to display cells with neuron-like characteristics, and ten days to obtain more than 70–80% of differentiated BM-MSC. Removing the neurogenic medium the differentiated BM-MSC did not spontaneously return to their fibroblastic morphology within the 48 hours. Due to stability of the differentiated state, we decided to analyze the profile of gene expression during the differentiating process.

Using the microarray technology a great number of genes can be compared in undifferentiated and differentiated BM-MSC and could explain the mechanism of neuronal differentiation. The protein expression profiles of MSC and osteoblasts revealed an important decreased expression of calmodulin, calponin, calpain, caldesmon and many others, after differentiation of MSC [32]. During the neuronal differentiation of BM-MSC, we observed similar decrease of mRNA levels corresponding to proteins involved in matrix mineralization. Interestingly, using our neuronal induction protocol, we also observed that genes implicated in neurogenesis are increased and specially those in relation with the synaptic transmission (AMPA, synaptotagmin, CASK, Syntaxin 1A, SNCAIP, MCT2). Indeed, each gene taken separately, we cannot conclude that BM-MSC can differentiate into neurons. But assembling all upregulated/downregulated genes, the differentiation potential of MSC into neural cell lineage seems confirmed. Analysing these results, we observed that genes implicated in neuronal differentiation of neuronal precursors, including SOCS2, sFRP1, amphiregulin, neuronal growth regulator 1 and EphA4, were also upregulated after neurogenic differentiation of BM-MSC [33-35]. More interestingly, in comparison of genes associated during neural differentiation process of human embryonic stem cells or human brain with our results, we also found increased expression of PENK, STK4, IGFBP4, Slc17a6, Slc6A1, Nurr1, Nrp2, Moxd1, Shh and Neurod2 [36,37]. Taken together with TH expression demonstrated by flow cytometry, we speculate that our neurogenic medium can promote the neuronal-dopaminergic differentiation pathway. Nevertheless, persistent high expression of nestin after 10 days of differentiation suggest the presence of mixed cell population at different stages of maturation or differentiation. However, the important decreased of genes involved in the proliferation suggests the post-mitotic state of these differentiated cells.

A recent study evaluating genes implied in neuronal differentiation of MSC, evidenced by Oligo GEarrays specially used to neural study (neurotransmitter receptors and regulators, neurogenesis and neural stem cells), demonstrated a large up-regulation of genes. More interestingly, as observed in our study, they also demonstrated the arrested growth of treated cells by important decrease of cell-cycle specific genes without increase of proapoptotic genes. They demonstrated also their functionality by the production of neurotransmitters and synaptic vesicle release of dopamine in response to a depolarizing stimulus [20].

Using similar neurogenic medium (NPMM or Neurobasal), MSC expressed dopaminergic specific genes (En1, En2, LMx1a, Wnt1, Ptx3, and Nuur1 and were positive to GFAP, MAP-2 and NSE but continued to express the neural stem cell marker nestin. Differentiated cells demonstrated the presence of two delayed rectifier K^+ ^currents and one Ca^2+^-depend K^+ ^regulatory currents contributing to the degree of excitability of neurons in human and produced neurotransmitter substance P with functional synapses. Nevertheless, treated cells were not always able to exhibit spontaneous and evoke action potential, suggesting that cells are not fully differentiated [38]. They confirmed our results by the fact that in vitro differentiation protocol could be sufficient to obtain post-mitotic neurons able to communicate with other cells by neurotransmitter way. Thus, it seems possible that BM-MSC may be primed *in vitro *towards mature dopamine secreting cells [20,39,40].

In our present study, microarray analysis and real-time PCR, realized both on BM-MSC and neuron-like cells, have demonstrated an increased expression of ionotropic glutamate receptor AMPA3, also called GRIA3 or GluR3. Functionality of this receptor was proved by measuring calcium influx in addition of specific agonists (AMPA or glutamate) or antagonist (CNQX). AMPA evoked an increased calcium influx in comparison with Calcium alone or in presence of antagonist (CNQX).

These recent finding cannot exclude the possibility that complete differentiation or maturation of differentiated cells require more specific factors. In fact, the microenvironment of each tissue plays an important role in the growth and differentiation of stem cells including local secreted factors, cell-cell interactions and extracellular matrix. It has been demonstrated that co-cultures with neurons or astrocytes are required for the functionality of BM-MSC derived neural cells [8,41,42]. After short co-culture period with neuro/glial cells, the neuro-mesenchymal cells were able to conduct single-action potentials as real neurons.

## Conclusion

In conclusion, the neurogenic induction protocol used in this study has no cytotoxic effect on MSC and promotes their differentiation into functional dopaminergic neurons as demonstrated by flow cytometry, RT-PCR, microarray and calcium influx. This medium will be important to guide their differentiation for future clinical uses in neurodegenerative diseases.

## Methods

### BM-MSC cultures

BM-MSC were obtained from healthy volunteer donors (n = 6) by sternal aspirates after informed consent. MSC were isolated using the classical adhesion method. Briefly, we seeded mononuclear cells (0,5.10^4 ^cells/cm^2^) in DMEM medium (Lonza, Verviers, Belgium) supplemented with 15% FBS (Sigma, St Louis, Missouri USA), 2 mM L-glutamine (Gibco BRL, Grand Island, NY) and 0,5% antibiotic-antimycotic solution (Gibco BRL). Cultures were replaced twice a week until 70–80% confluent. BM-MSC were characterized by flow cytometry and their differentiation potential was investigated. Adipogenic, osteogenic and chondrogenic differentiation was performed as previously described [17]. Lipid vacuoles, calcium deposits and chondrogenic matrix were observed and identified by Oil Red O (Sigma), Von Kossa, and toluidin blue staining methods respectively.

### Antibodies and cytokines

The MSC phenotype was determined using the following antibodies: CD34-PE/CY5 (ImTec Diagnostics NV), CD45-CY5 (BD Pharmingen, Erembodegem, Belgium), CD73-PE (BD), CD90-FITC (RD System, Abingdon, United Kingdom), CD105-FITC (RD System), CD166-FITC (AbD Serotec, Cergy St Christophe, France) and HLA-Dr-CY5 (Immunotech, Marseille, France). Cells were incubated during 30 minutes at room temperature. Data were acquired on a Coulter EPICS-XL (Coulter, Miami, FL, USA).

### Neurogenic differentiation

After 2 passages, when cells are positive for mesenchymal markers (CD105, CD90, CD73, CD166) and negative for hematopoietic/endothelial markers (HLA-Dr, CD34, CD45), we differentiated them with the neurogenic medium as previously described [14]. Briefly, 1,5.10^3 ^cells/cm^2 ^were cultured in NPBM medium (Cambrex). This medium was supplemented with 5 μM cAMP (Sigma), 5 μM IBMX (Sigma), 25 ng/mL NGF (Sigma) and 2,5 μg/mL insuline (Sigma). Half differentiation medium was changed twice a week during ten days. Three undifferentiated/differentiated cell couples were used for the microarray study and three new couples were added for real-time PCR analysis.

### Flow cytometric analysis of differentiated cells

The effect of our neurogenic medium on neuronal marker expression was evaluated by flow cytometric analysis using the following antibodies: nestin (Chemicon International, Temecula, CA USA), MAP-2 (Sigma) and TH (Sigma). Since these proteins were intracytoplasmic, cells were fixed and permeabilized with an acetone/methanol (1/1 solution) for 5 min at -20°C.

### Microarray analysis

Total RNA from both differentiated and undifferentiated MSC was extracted and purified with RNeasy purification Kit (Qiagen, Valencia, CA, USA). Then, double-stranded cDNA was synthesized using the One-Cycle cDNA synthesis Kit (Affymetrix, Santa Clara, CA, USA). The cRNA, performed by in vitro transcription, was biotinylated using the IVT Labeling Kit according to the manufacturer's recommendations (Affymetrix) and hybrided to the probe HG-U133 Plus 2.0 GeneChips which included 54675 cDNA sequences (Affymetrix). Genes called "absent" for six or five out of six MSC (undifferentiated/differentiated), defined by the Affymetric algorithm, were excluded. Data were normalized with RMA method, with processes a group of CEL files simultaneously [18]. The signal intensity with a fold change of 1,5 (decrease or increase)and common to each group of differentiated/undifferentiated MSC were analyzed using the parametric t-test with a p-value <0,05 and a fold discovery rate (FDR) of q<7%. We identified 1943 genes in which 913 were upregulated and 1030 downregulated after neurogenic differentiation.

### Quantitative Real-Time PCR analysis

Total RNA was extracted from differentiated and undifferentiated MSC using Tripure Isolation Reagent (Roche Applied Science, Mannheim, Germany). We performed the reverse transcription with 1 μg RNA using the M-MLV reverse transcriptase (Invitrogen life Technologies, Merelbeek, Belgium) in a final volume of 20 μL containing 1 μg RNA, 4 μL first strand buffer, 10^-2 ^M DTT, 2 mM of each dNTP, 12 U Rnase inhibitor and 100 U M-MLV reverse transcriptase. The reverse transcription was performed for 45 minutes at 42°C and followed by an enzyme-inactivation step. Then, transcripts encoding neuro-regulatory or mesenchymal proteins (Table 1) [see additional file 1] were quantified using 20 ng of cDNA in a quantitative PCR with 2X SYBR Green PCR Master Mix (Applied Biosystems, Lennik, Belgium) added with 0,32 μM of each forward and reverse primers. These primers were designed with the Primer Express 2.0 software (Applied Biosystems). To compensate variation of input RNA amount or efficiency of reverse transcription enzyme, a housekeeping gene was used to quantify and normalize the results (β-actin). The reaction was carried out using the ABI Prism 7900 HT (Applied Biosystems). In all cases, dissociation curves were made and confirmed the specificity of PCR reactions. The amplified fragments were isolated (Qiaquick Gel Extraction kit, Qiagen) and diluted to generate the standard curves for reproducibility and efficiency of the reaction. The comparative ct method was applied for data analysis.

### Ca2+ influx measurement

Intracellular fluorescent Ca2+ was measured with Fluo-4 indicator (Molecular Probes, Belgium) in neuron-like cells cultured in 96 well black microplates (Poly D-lysin coated, Greiner Bio-One, Germany). Cells were seeded at 3000 cells/well during 48 hours and then differentiated during 10 days with the neurogenic medium. Briefly, Fluo-4 solution (2,5 mM) was prepared according the manufacturer's recommendations and put on cell culture. Plates were first incubated at 37°C for 30 minutes and then at room temperature for an additional time of 30 minutes. Then, we added CNQX (10 μM, 6-cyano-7nitroquinoxaline-2,3-dione, Sigma), (±) AMPA hydrobromide (40 μM, α-Amino-3-hudroxy-5-methylisoxazole-4-propionic acid, Sigma) or Glutamate (100 μM, Sigma) in a calcium buffer solution containing 5 mM CaEGTA, 5 mM EGTA, 100 mM KCl and 30 mM MOPS with free 0.15 μM Ca^2+ ^(Molecular Probes) and plates were measured immediately.

The Fluo-4 indicator was excited at 488 nm and analyzed at 518 nm using the Fluostar optima (BMG Labtech, Netherlands). Measures were acquired every 4 second interval and expressed as Fluorescence Intensity.

## Supplementary Material

Additional file 1 Tables. Primer sequences used for qRT-PCR and gene list detected by microarray. Click here for file

## References

[B1] Kelly S, Bliss TM, Shah AK, Sun GH, Ma M, Foo WC, Masel J, Yenari MA, Weissman IL, Uchida N, Palmer T, Steinberg GK (2004). Transplanted human fetal neural stem cells, survive, migrate, and differentiate in ischemic rat cerebral cortex. PNAS.

[B2] Takagi Y, Nishimura M, Morizane A, Takashashi J, Nozaki K, Hayashi J, Hashimoto N (2005). Survival and differentiation of neural progenitor cells derived from embryonic stem cells and transplanted into ischemic brain. J Neurosurg.

[B3] Hofstetter CP, Schwarz EJ, Hess D, Widenfalk J, Manira El A, Prockop DJ, Olson L (2002). Marrow stromal cells from guiding strands in the injured spinal cord and promote recovery. PNAS.

[B4] Li Y, Chen J, Wang L, Zhang L, Lu M, Chopp M (2001). Intracerebral transplantation of bone marrow stromal cells in a 1-methyl-4-phenyl-1,2,3,6-tatrahydropyridine mouse model of Parkinson's disease. Neurosci Lett.

[B5] Azizi SA, Stokes D, Augelli BJ, DiGirolamo C, Prockop DJ (1998). Engraftment and migration of bone marrow stromal cells implanted in the brain of albino rats-similarities to astrocyte grafts. PNAS.

[B6] Crigler L, Robey RC, Asawachaicham A, Gaupp D, Phinney DG (2006). Human mesenchymal stem cell subpopulations express a variety of neuro-regulatory molecules and promote neuronal survival and neuritogenesis. Exp Neurol.

[B7] S Wislet-Gendebien, Hans G, Leprince P, Rigo JM, Moonen G, Rogister B (2005). Plasticity of cultured mesenchymal stem cells: switch from nestin-positive to excitable neuron-like phenotype. Stem Cells.

[B8] Abouelfetouh A, Kondoh T, Ehara K, Kohmura E (2004). Morphological differentiation of bone marrow stromal cells into neuron-like cells after co-culture with hippocampal slice. Brain Res.

[B9] Woodbury D, Schwarz EJ, Prockop DJ, Black IB (2000). Adult rat and human bone marrow stromal cells differentiate into neurons. J Neurosci Res.

[B10] Deng W, Obrocka M, Fischer I, Prockop DJ (2001). In vitro differentiation of human marrow stromal cells into early progenitors of neural cells by conditions that increase intracellular cyclic AMP. Biochem Biophys Res Commun.

[B11] Neuhuber B, Gallo G, Howard L, Kostura L, Mackay A, Fischer I (2004). Reevaluation of in vitro differentiation protocols for bone marrow stromal cells: disruption of actin cytoskeleton induces rapid morphological changes and mimics neuronal phenotype. J Neurosci Res.

[B12] Lu P, Blesch A, Tuszynski MH (2004). Induction of bone marrow stromal cells to neurons: differentiation, transdifferentiation, or atifact?. J Neurosci Res.

[B13] Choi CB, Cho YK, Prakash KV, Jee BK, Han CW, Paik YK, Kim HY, Lee KH, Chung N, Rha HK (2006). Analysis of neuron-like differentiation of human bone marrow mesenchymal stem cells. Biochem Biophys Res Commun.

[B14] Tondreau T, Lagneaux L, Dejeneffe M, Massy M, Mortier C, Delforge A, Bron D (2004). Bone marrow-derived mesenchymal stem cells already express specific neural proteins before any differentiation. Differentiation.

[B15] Mareschi K, Novara M, Rustichelli D, Ferrero I, Guido D, Carbone E, Medico E, Madon E, Vercelli A, Fagioli F (2006). Neural differentiation of human mesenchymal stem cells: Evidence of neuronal markers and eag K^+ ^channel types. Exp Hematol.

[B16] Long X, Olszewiski M, Huang W, Kletzel M (2005). Neural cell differntiaiton in vitro from adult human bone marrow mesenchymal stem cells. Stem Cells Dev.

[B17] Tondreau T, Lagneaux L, Dejeneffe M, Delforge A, Massy M, Mortier C, Bron D (2004). Isolation of bone marrow mesenchymal stem cells by plastic adhesion or negative selection : phenotype, proliferation kinetics and differentiation potential. Cytotherapy.

[B18] Bolstad BM, Irizarry RA, Anstrand M, Speed TP (2003). A comparison of normalization for high density oligonucleotide array data based on variance and v-bias. Bioinformatics.

[B19] Egusa H, Schweizer FE, Wang CC, Matsuka Y, Nishimura I (2005). Neuronal differentiation of bone marrow-derived stromal stem cells involves suppression of discordant phenotypes through gene silencing. Journal of Biology and Chemistry.

[B20] Greco SJ, Zhou C, Ye J-H, Rameshwar P (2007). An interdiscipliary approach and characterization of neuronal cells transdifferentiated from human mesenchymal stem cells. Stem Cells Dev.

[B21] Silva WA, Covas DT, Panepucci RA, Proto-Siqueira R, Siufi JLC, Zanette DL, Santos ARD, Zago MA (2003). The profile of gene expression of human marrow mesenchymal stem cells. Stem Cells.

[B22] Bennett CN, Longo KA, Wright WS, Suva LJ, Lane TF, Hankenson KD, MacDougald OA (2005). Regulation of osteogenesis and bone mass by Wnt10b. PNAS.

[B23] Keating A (2006). Mesenchymal stromal cells. Curr Opin Hematol.

[B24] Munoz-Elias G, Woodbury D, Black IR (2003). Marrow stromal cells, mitosis, and neuronal differentiation: stem cells and precursor functions. Stem Cells.

[B25] Suzuki H, Taguchi T, Tanaka H, Kataoka H, Li Z, Muramatsu K, Gondo T, Kawai S (2004). Neurospheres induced from bone marrow stromal cells are multipotent for differentiation into neuron, astrocyte and oligodendrocyte phenotype. Biochem Biophys Res Commun.

[B26] Bertani N, Malatesta P, Volpi G, Sonego P, Perris R (2005). Neurogenic potential of human mesenchymal stem cells revisited: analysis by immunostaining, time-lapse video and microarray. J Cell Sci.

[B27] Krabbe C, Zimmer J, Meyer M (2005). Neural transdifferentiation of MSC–a critical review. APMIS.

[B28] Tremain N, Korkko J, Ibberson D, Kopen GC, DiGiramolo C, Phinney DG (2001). MicroSAGE analysis of 2353 expressed genes in a single cell-derived colony of undifferentiated human mesenchymal stem cells reveals mRNAs of multiple cell lineages. Stem Cells.

[B29] Woodbury D, Reynolds K, Black IB (2002). Adult bone marrow stromal stem cells express germline, ectodermal, endodermal, and mesodermal prior to neurogenesis. J Neurosci Res.

[B30] Deng J, Pertersen BE, Steindler DA, Jorgensen ML, Laywell ED (2006). Mesenchymal stem cells spontaneously express neural proteins in culture and are neurogenic after transplantation. Stem Cells.

[B31] Suon S, Jin H, Donaldson AE, Caterson EJ, Tuan RS, Deschennes G, Marshall C, Lacovitti L (2004). Transient differentiation of adult human bone marrow cells into neuron like cells in culture: development of morphological and biochemical traits is mediated by different molecular mechanisms. Stem Cells Dev.

[B32] Salasznyk RM, Westcott AM, Klees RF, Ward DF, Xiang Z, Vandenberg S, Benett K, Lopper PGE (2005). Comparing the protein expression profiles of human mesenchymal stem cells and human osteoblasts using gene ontologies. Stem Cells Dev.

[B33] Falk A, Frisen J (2002). Amphiregulin is a mitogen for adult neural stem cells. J Neurosci Rese.

[B34] Goldshmit Y, Greenhalgh CJ, Turnley AM (2004). Suppressor of cytokine signalling-2 and epidermal growth factor regulate neurite outgrowth of cortical neurons. Eur J Neurosci.

[B35] Augustine C, Gunnersen J, Spirkoska V, Tan SS (2001). Place-and time-development of the cerebral neocortex. Mech Dev.

[B36] Anisimov SV, Christophersen NS, Correia AS, Li J-Y, Brundin P (2007). "NeuroStem Chip": a novel hoghlu specialized tool to study neural differentiation pathways in human stem cells. BMC Genomics.

[B37] Yu S, Zhang JZ, Xu Q (2006). Genes associated with neuronal differentiation of precursors from humain brain. Neurosci.

[B38] Trzaska KA, Kulzhikandathil EV, Rameshwar P (2007). Specification of Dopaminergic phenotype from adult human mesechymal stem cells. Stem Cells.

[B39] Cho KJ, Trzaska KA, Greco SJ, McArdle J, Wang FS, Ye JH, Rameshwar P (2005). Neurons Derived From Human Mesenchymal Stem Cells Show Synaptic Transmission and Can Be Induced to Produce the Neurotransmitter Substance P by Interleukin-1. Stem cells.

[B40] Kan I, Ben-Zur T, Barhum Y, Levy YS, Burstein A, Charlow T, Bulvik S, Melamed E, Offen D (2007). Dopaminergic differentiation of human mesenchymal stem cells-Utilization of bioassay for tyrosine hydroxylase expression. Neurosci Lett.

[B41] Joannides A, Gaughwin P, Schwiening C, Majed H, Sterling J, Campston A, Chandran S (2004). Efficient generation of neural precursors from adult human skin: astrocytes promote neurogenesis from skin-derived stem cells. The Lancet.

[B42] Bossolasco P, Cova L, Calzarossa C, Rimoldi SG, Borsotti C, Lambertenghi G, Silani V, Soligo D, Polli E (2005). Neuro-glial differentiation of bone marrow stem cells in vitro. Exp Neurol.

