# Supplementary material for: Gene expression pattern of functional neuronal cells derived from human bone marrow mesenchymal stromal cells
Source: BMC Genomics. 2008 Apr 11;9:166. doi: 10.1186/1471-2164-9-166 (PMC2358905; doi:10.1186/1471-2164-9-166)
Supplement: Additional file 1 — Tables. Primer sequences used for qRT-PCR and gene list detected by microarray. [file 1471-2164-9-166-S1.doc]

**Table 1** :Primer sequences used to validate microarray results by quantitative real-time PCR.

|  | **forward 5'-3'** | **reverse 5'-3'** | **Gene Access** |
| --- | --- | --- | --- |
| **bActin** | ctg gca ccc agc aca atg | ccg atc cac acg gag tac ttg | NM_001101 |
| **NEGR1** | tca ttc cca gat gtg agg aaa gta a | ggt cac ggt gcc aga ttt aat t | NM_173808 |
| **EPHA4** | gtg agc cct tgg agg tta caa c | act gtg gag tta gcc cca tct c | NM_004438 |
| **NEF3** | tag aaa tcg ctg cgt aca gaa aac | tgc ttc ctg caa atg tgc taa | NM_005382 |
| **PENK** | tcc tgg ctt gcg taa tgg a | ctc ctt gca ggt ttc cca aa | NM_006211 |
| **GRIA3** (AMPA3) | aca cca tca gca tag gtg gac tt | acg gca aag cgg aaa gc | BC032004 |
| **HAPNL1** (Hyaluronan**)** | gat act gtt gtg gta gca ctg gac tt | cgc ccc agt cgt gga a | NM_001884 |
| **VIM** (Vimentine) | atg ccc tta aag gaa cca atg ag | caa cgg caa agt tct ctt cca t | NM_003380 |
| **CSPG4** | gga ggc cct ggt gaa ctt c | ggc atc tca tgc tca tac aga tat t | NM_001897 |
| **CALD1** | aga aaa gca gtg gtg tca aat cg | cca gtc tgc tgt caa tct tgg a | BC040354 |
| **DSC96** | tcc ata atg aag ccc tgt tgt gt | tag gag ggc ccc caa gaa | AF242771 |
| **MEST** | tcg agg tct cac ccc agt ctt | cca cat gtc cca cag ctc act | D78611 |
| **TUFT1** | aga gcc agc agc gga aag t | ttg act gga tca cag ctt ttg aa | NM020127 |

NEGR1:Neuronal Growth Regulator 1, EPHA4: Ephrin receptor A4, NEF3: Neurofilament 3, PENK: Proenkephalin, GRIA3 (AMPA3): Glutamate receptor, ionotropic AMPA3, HAPNL1: Hyaluran and proteoglycan link protein 1, VIM: Vimentin, CSPG4: Chondroitin Sulfate Proteoglycan 4, CALD1: Caldesmon, DSC96:Mesenchymal stem cell protein DSC96, MEST: Mesoderm specific transcript, TUFT1: Tuftelin.

**Table 2**: List of genes detected by microarray analysis and modification of their expression after neurogenic differentiation (undifferentiated versus differentiated MSC)

| **Gene name** | **Gene symbol** | **Fold Change** | **p-value** |
| --- | --- | --- | --- |
|  |  |  |  |
| **Neuronal Channel/Transporter** |  |  |  |
| Potassium channel, subfamily K, member 1 | KCNK1 | 4,3 | 0,00012 |
| Potassium voltage gated channel, subfamily G, member 1 | KCNG1 | 3,1 | 0,00013 |
| Solute carrier family 22, member 17 | SLC22A17 | 3,1 | 0,00016 |
| Solute carrier family 7, member 8 (LAT2) | SLC7A8 | 3,3 | 0,00016 |
| Potassium inwardly-rectifying channel, subf.J, member 8 | KCNJ8 | 2,3 | 0,00019 |
| Solute carrier family 19 member 4 (MCT4) | SLC16A4 | 4,4 | 0,00033 |
| Solute carrier family 16, member 6 (MCT6) | SLC16A6 | 78,9 | 0,0012 |
| Chloride Channel 3 | CLCN3 | 2,4 | 0,002 |
| Potassium inwardly-rectifying channel, subf. J, member 2 | KCNJ2 | 16,1 | 0,0081 |
| Potassium inwardly-rectifying channem, subf. J, member 15 | KCNJ15 | 3,5 | 0,0083 |
|  |  |  |  |
| **Synaptic Differentiation/Transmission** |  |  |  |
| Cortactin | CTTN | 3,2 | 0,00015 |
| Pro-melanin-concentrating hormone | PMCH | 102,7 | 0,00035 |
| Phosphodiesterase E4 | PDE4B | 15,9 | 0,00052 |
| Proprotein convertase subtilisin/kexin type 1 | PCSK1 | 110,2 | 0,00059 |
| Calcium/calmodulin-dependent serine protein kinase | CASK | 2,1 | 0,00067 |
| Glutamate receptor, ionotrophic, AMPA3 | GRIA3 | 2,9 | 0,0017 |
| Synaptotagmin binding, cytoplasmic RNA interacting protein | SYNCRIP | 1,7 | 0,0019 |
| Golgi transport 1 homolog B | GOLT1B | 3 | 0,0019 |
| Synaptotagmin-like 4 | SYTL4 | 7,9 | 0,003 |
| Fibroblast growth factor 7 | FGF7 | 4,9 | 0,00018 |
| Rho GTPase activating protein 6 | ARHGAP6 | 23,2 | 0,00468 |
|  |  |  |  |
| **Neuronal Development** |  |  |  |
| Secreed frizzled related protein 1 | SFRP1 | 25,2 | 0,00014 |
| Neuronal growth regulator 1 | NEGR1 | 6,3 | 0,00041 |
| Brain derived neurotrophic factor | BDNF | 3,9 | 0,0006 |
| Acyl-CoA Synthetase long chain family member 3 | ACSL3 | 2,1 | 0,00062 |
| Sonic hedgehog | Shh | 2,5 | 0,0007 |
| Neuropilin (BRP) and tolloid (TLL)-like 2 | NETO2 | 34,5 | 0,0012 |
| Integral membrane protein 2B | ITM2B | 2,3 | 0,0016 |
| Low density lipoprotein receptor-related protein 8, apolipoprotein e receptor | LRP8 | 5,6 | 0,0023 |
| Neuropilin 2 | NRP2 | 1,6 | 0,0026 |
| Sphingomyelin phophodiesterase 1, acid lysosomial (acide sphingomyelinase) | SMPD1 | 2 | 0,0027 |
| Brain abundant, membrane attached signal protein 1 | BASP1 | 1,8 | 0,0031 |
| CDK5 regulatory subunit associated protein 2 | CDK5RAP2 | 2 | 0,0032 |
| Laminin, alpha 3 | LAMA3 | 20,8 | 0,0049 |
| Monoaxygenase, DBH-like 1 | MOXD1 | 2,6 | 0,0046 |
| Glutamate-cystein ligase, modifier subunit | GCLM | 1,9 | 0,0058 |
| GABA(A) receptor associated protein-like 1 | GABARAPL1 | 2 | 0,0067 |
| Synuclein, alpha interacting protein (synphilin) | SNCAIP | 3,1 | 0,0074 |
| Bone morphogenic protein 6 | BMP6 | 11,9 | 0,0112 |
| Syntaxin 1A | STX1 | 5 | 0,0153 |
| Insuline-like growth factor 1 | IGF-1 | 4.4 | 0.013 |
|  |  |  |  |
| **Other neuronal genes** |  |  |  |
| FK506 binding protein 7 | FKBP7 | 3,2 | 0,00016 |
| EPH receptor A4 | EPHA4 | 2,9 | 0,00048 |
| Ly6/neurotoxin1 | LYNX1 | 2,7 | 0,0013 |
| Neurofilament 3 (150kDa) | NEF3 | 4,6 | 0,0014 |
| Galactosamine:polypeptide N-acetylgalactosaminyltransferase-like 2 | GALNTL2 | 18,5 | 0,0015 |
| Glutamine-fructose-6-phosphate transaminase 2 | GFPT2 | 8,1 | 0,0019 |
| Ets variant gene 1 | ETV1 | 6 | 0,0024 |
| Proenkephalin | PENK | 3,1 | 0,0035 |
| Fatty acid desaturase | FADS | 3,3 | 0,0039 |
| Amphiregulin (schwannoma-derived growth factor) | AREG | 100,6 | 0,0043 |
| Phospholipase D 1 | PLD1 | 2,8 | 0,004 |
| Acyl-CoA synthetase long-chain family member 4 | ACSL4 | 9,8 | 0,0098 |
| Fatty acid synthase | FASN | 2,9 | 0,005 |
| Insulin like growth factor binding protein 4 | IGFBP4 | 2,3 | 0,011 |
| Suppressor of cytokine signaling 2 | SOCS2 | 14 | 0,0144 |
| G-protein coupled receptor 125 | GPR 125 | 5,8 | 0,0158 |
| Aldo-keto reductaze family 1, member C1 | AKR1C1 | 6,9 | 0,014 |
|  |  |  |  |
| **Mesodermal Differentiation** |  |  |  |
| CHONDROGENESIS |  |  |  |
| Tensin | TNS | -3,5 | 0,00009 |
| Hyaluronan and proteoglycan link protein 1 | HAPLN1 | -19,6 | 0,00015 |
| Large aggregating proteoglycan | AGC1 | -8 | 0,0004 |
| Collagen, type XI, alpha 1 | COL11A1 | -14,7 | 0,0022 |
|  |  |  |  |
| OSTEOGENESIS |  |  |  |
| PDZ and LIM domain 7 | PDLIM7 | -4,7 | 0,00022 |
| Tuftelin | TUFT1 | -3,9 | 0,00081 |
| Palladin | KIAA0992 | -4,4 | 0,0021 |
| Biglycan | BGN | -4 | 0,0023 |
| Gremlin 2 | GREM2 | -3,7 | 0,0032 |
|  |  |  |  |
| MYOGENESIS |  |  |  |
| Syncoilin, intermedoiadte filament 1 | SYNC1 | -4,8 | 0,00027 |
| Tropomyosin 1 (alpha) | TPM1 | -9,2 | 0,00035 |
| Tropomyosin 2 (beta) | TPM2 | -3,4 | 0,00041 |
| Glycogen synthase 1 | GYS1 | -2,3 | 0,00045 |
| Caldesmon 1 | CALD1 | -4,6 | 0,00053 |
| Actin, alpha | ACTC | -4,2 | 0,00055 |
| Desmuslin | DMN | -4,1 | 0,00057 |
| MADS box transcription enhancer factor 2 polypeptide A | MEF2A | -2,3 | 0,00091 |
| Synaptopodin 2 | SYNPO2 | -43,4 | 0,0011 |
| Leiomodulin | LMOD1 | -7,5 | 0,0176 |
| Myosin | MYL9 | -5,4 | 0,0038 |
|  |  |  |  |
| ADIPOGENESIS |  |  |  |
| Fatty acid desaturase 3 | FADS3 | -2,1 | 0,00035 |
| Monoglyceride lipase | MGLL | -3,1 | 0,0011 |
| Lysophospholipase-like 1 | LYPLAL1 | -1,6 | 0,0025 |
|  |  |  |  |
| **Extracellular Matrix Component** |  |  |  |
| Chondroitin sulfate proteoglycan 4 | CSPG4 | -2,1 | 0,00088 |
| Microfibrillar associated protein 5 | MFAP5 | -30,7 | 0,0013 |
| Extracellular matrix protein 2 | ECM2 | -10,7 | 0,0021 |
| Collagen, type VIII, alpha 2 | COL8A2 | -3,3 | 0,0025 |
| Hyaluronan synthase 3 | HAS3 | -3,8 | 0,0079 |
| Syndecan 2 | SDC2 | -4 | 0,0088 |
| Matrix remodelling associated 5 | MXRA5 | -15,9 | 0,0164 |
|  |  |  |  |
| **Cytoskeleton** |  |  |  |
| Tubulin, beta 6 | TUBB6 | -3 | 0,00015 |
| Tubulin, beta 2 | TUBB2 | -2,7 | 0,00027 |
| Tubulin, alpha 6 | TUBA6 | -1,8 | 0,00088 |
| Filamin A, alpha | FLNA | -3,4 | 0,00095 |
| Tubulin, beta | TUBB3 | -2,5 | 0,0011 |
| Vimentin | VIM | -3,4 | 0,02385 |
|  |  |  |  |
| **Cell cycle/Proliferation** |  |  |  |
| Cell division cycle 2, G1 to S and M | CDC2 | -7,6 | 0,00024 |
| Fibroblast growth factor receptor 2 | FGFR2 | -11,2 | 0,0008 |
| CDC42 effector protein | CDC42EP3 | -9,6 | 0,00097 |
| TPX microtubule-associated | TPX2 | -6 | 0,0012 |
| S-phase response (cyclin-related) | SPHAR | -2,2 | 0,0017 |
| Cell cycle progression 1 | CCPG1 | -1,7 | 0,0035 |
| Cyclin A2 | CCNA2 | -3,9 | 0,005 |
| Cyclin B2 | CCNB2 | -4 | 0,0059 |
| Glypican 4 | GPC4 | -4,2 | 0,00247 |
|  |  |  |  |
| **Others mesodermal genes** |  |  |  |
| Platelet-derived growth factors alpha polypeptide | PDGFA | -2,5 | 0,00032 |
| SLC2A4 regulator (GLUT4) | SLC2A4RG | -2,8 | 0,0024 |
| Mesenchymal stem cell protein DSC96 | DSC96 | -5,8 | 0,0044 |
| Insulin-like growth factor binding protein 7 | IGFBP7 | -2,3 | 0,0053 |
| PRKC, apoptosis, WT1, regulator | PAWR | -2,3 | 0,00326 |
| Collagen type 1, alpha1 | COL1A1 | -8,1 | 0,0068 |
| Mesoderm specific transcript | MEST | -15,1 | 0,0205 |
